# Interferon Receptor Trafficking and Signaling: Journey to the Cross Roads

**DOI:** 10.3389/fimmu.2020.615603

**Published:** 2021-01-20

**Authors:** Natacha Zanin, Christine Viaris de Lesegno, Christophe Lamaze, Cedric M. Blouin

**Affiliations:** ^1^ NDORMS, The Kennedy Institute of Rheumatology, University of Oxford, Oxford, United Kingdom; ^2^ Institut Curie—Centre de Recherche, PSL Research University, Membrane Dynamics and Mechanics of Intracellular Signalling Laboratory, Paris, France; ^3^ Institut National de la Santé et de la Recherche Médicale (INSERM), Paris, France; ^4^ Centre National de la Recherche Scientifique (CNRS), UMR 3666, Paris, France

**Keywords:** transmembrane receptor, interferon, endocytosis, intracellular signaling, traffic, JAK - STAT signaling pathway

## Abstract

Like most plasma membrane proteins, type I interferon (IFN) receptor (IFNAR) traffics from the outer surface to the inner compartments of the cell. Long considered as a passive means to simply control subunits availability at the plasma membrane, an array of new evidence establishes IFNAR endocytosis as an active contributor to the regulation of signal transduction triggered by IFN binding to IFNAR. During its complex journey initiated at the plasma membrane, the internalized IFNAR complex, i.e. IFNAR1 and IFNAR2 subunits, will experience post-translational modifications and recruit specific effectors. These finely tuned interactions will determine not only IFNAR subunits destiny (lysosomal degradation vs. plasma membrane recycling) but also the control of IFN-induced signal transduction. Finally, the IFNAR system perfectly illustrates the paradigm of the crosstalk between membrane trafficking and intracellular signaling. Investigating the complexity of IFN receptor intracellular routes is therefore necessary to reveal new insight into the role of IFNAR membrane dynamics in type I IFNs signaling selectivity and biological activity.

## Introduction

The IFNAR signaling pathway plays a central role in the defenses of the organism by supporting one of the major anti-viral and anti-proliferative cellular responses. Its dysregulation can also lead to deleterious auto-inflammation in humans (1). Nowadays, it is accepted that endocytosis holds an essential role in the activity of a large number of receptors including receptor tyrosine kinases (RTK) and G protein-coupled receptors (GPCR) families [reviewed in (2, 3)]. The role of endocytosis in the modulation of type I interferons receptor (IFNAR) has however lagged behind. Endocytosis is an essential mechanism by which a cell can efficiently achieve the uptake of transmembrane proteins, lipids, nutrients, extracellular molecules, and more generally cell surface cargos. The identification and characterization of clathrin-coated pits shed light upon endocytosis as being an active and highly regulated process mediated by clathrin and dynamin (4–6). Over the last decades, a strong body of work has contributed to the complexification of the mechanisms involved in the regulation of endocytosis. Early on, endocytosis was categorized as being mediated either by clathrin-dependent or clathrin-independent means. This simplistic binary classification did not resist further investigations and today several molecular machineries including caveolin, endophilinA2, RhoA, Cdc42, Arf6, flotillin or endophilinA3/galectin8 selectively control distinct endocytic pathways (7–9). Despite the existence of specific molecular machineries, these different endocytic pathways share the common property that is to modulate the cell surface density of a multitude of receptors, a process that is essential for the cell homeostasis and the transduction of receptor signaling (10, 11). Whereas the IFNAR signaling cascade has long been thought to be linear and exclusively controlled at the plasma membrane (PM) [reviewed in (12)], these studies allow to revisit its regulation in the context of membrane trafficking. In this review, we will describe recent studies on IFNAR journey from the cell surface to the different endosomal compartments and how it is connected with the regulation of signaling outputs.

## Mechanisms That Control Steady State Type I Interferon Receptor Levels

The type I IFN receptor is composed of the IFNAR1 and IFNAR2 subunits. IFNAR1 exists only as one isoform whereas differential splicing of the *IFNAR2* gene generates three isoforms. The firstly discovered IFNAR2c is the longer isoform with the full intracellular domain (13). IFNAR2b is a shorter transmembrane isoform lacking the intracellular domain while IFNAR2a is a soluble truncated form. The two latter isoforms can still bind type I IFNs and interact with IFNAR1 but are unable to transduce signal (14), suggesting they would be negative regulators of JAK/STAT signaling (15). We will focus here only on the full-length IFNAR2c which will be referred to as IFNAR2.

IFNAR1 and IFNAR2 are ubiquitously expressed (16) albeit with highly variable levels. The first quantifications of PM levels relied on standard Scatchard analysis, which is based on the saturation of IFNAR binding with iodinated IFNs and allow to precisely determine ligand affinity and number of binding sites (17). Published results showed large variations among cell types with a number of binding sites ranging from 200 to up to 250,000 (18, 19). Today, Scatchard analysis has been replaced by more acute and sophisticated measurements. For instance, single-molecule imaging of fluorescently labelled IFN-α2 by total internal reflection fluorescence (TIRF) microscopy could measure a density of around 0.55 IFN bound per μm² in HeLa cells, corresponding to 500–1000 binding sites per cell (20). The same technique measured 0.58 IFNAR1 and 0.72 IFNAR2 per μm² of PM in human retinal pigmented epithelial RPE1 cells (21). While the IFNAR cytoplasmic pool is likely to be important, few if any studies have determined the ratio of internal versus surface IFNAR. Likewise, the PM IFNAR1/IFNAR2 ratio is certainly critical for IFNAR signal transduction. Finally, the two IFNAR2a and b shorter forms can compete with the long IFNAR2c by forming a non-signaling complex (14).

### Type I Interferon Receptor Intimate Cytosolic Interactors

**Figure 1:**
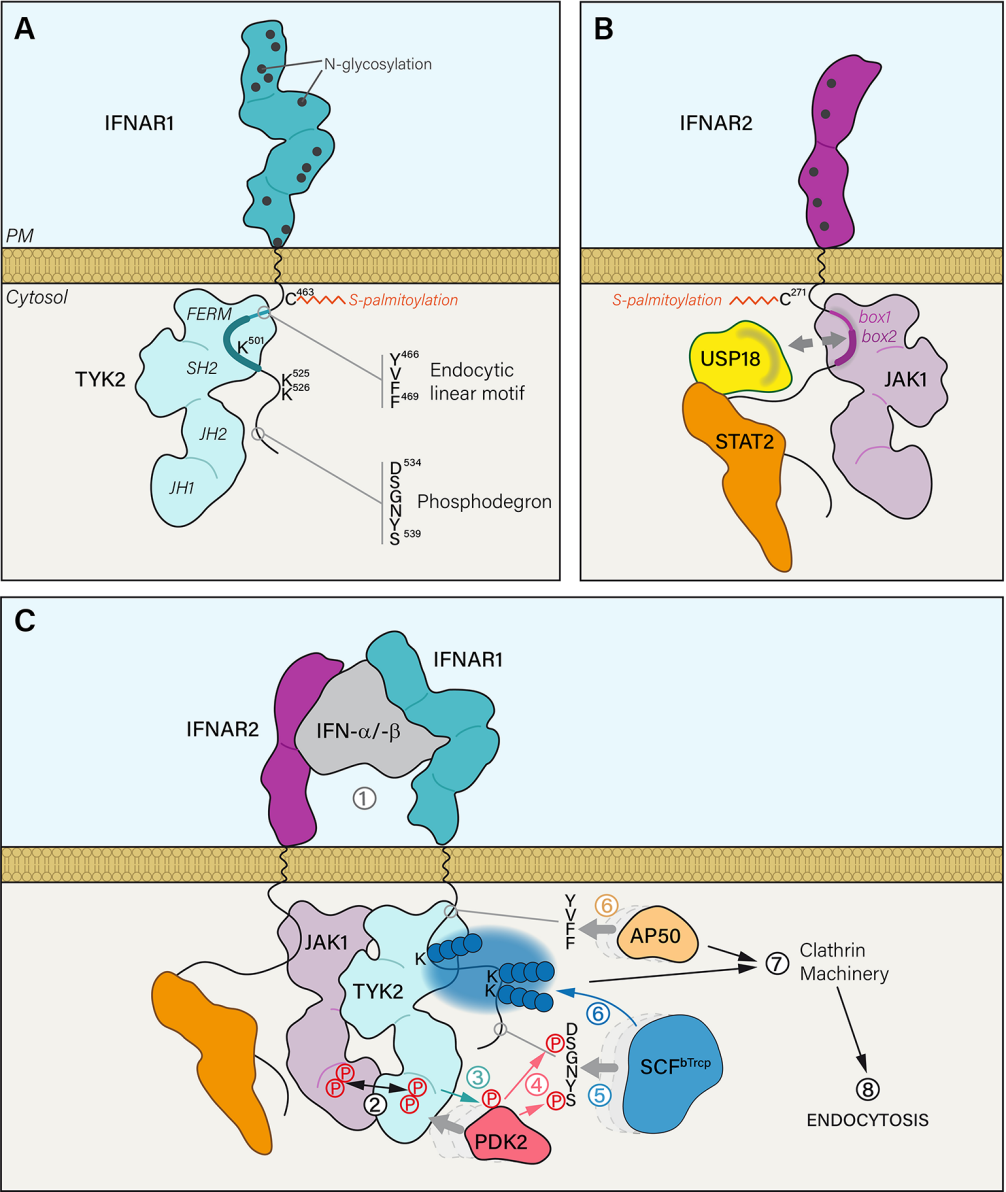
**(A)** The extracellular part of IFNAR1 is composed of four domains and exhibits 12 residues that are potentially N-glycosylated. IFNAR1 is S-palmitoylated on the PM proximal Cys^463^ residue. IFNAR1 cytosolic tail interacts with TYK2 kinase FERM and SH2 domains through a minimal region that corresponds to 486–511 residues (thick blue line). IFNAR1 interaction domain with TYK2 can be extended to a maximal region from residues 465 to 511 (thick and thin blue line) that also covers a canonical tyrosine-based linear endocytic motif ^466^YVFF^469^. IFNAR1 cytosolic tail has three lysine residues that can be ubiquitinated and a phosphodegron motif. **(B)** The extracellular part of IFNAR2 is composed of two domains presenting five putative N-glycosylation sites. IFNAR2 is meant to be S-palmitoylated on two Cys residues: one near the PM (Cys^271^) and another one less likely, closer to the C-terminal part (Cys^395^). IFNAR2 interacts with its associated JAK1 kinase through cytosolic tail box 1 and box 2 domains. At steady state, ubiquitin specific peptidase 18 (USP18) interacts with the DNA-binding and coiled-coil domains of STAT2 but also with IFNAR2 box 1 and box 2 domains. Therefore, it can compete with JAK1 binding on IFNAR2. **(C)** (1) IFN-α/-β binding to IFNAR1 and IFNAR2 subunits triggers several mechanistic events leading to the internalization of the receptor complex. (2) IFNAR associated JAK kinases are brought in close proximity resulting in the concomitant tyrosine phosphorylation of JAK1 and TYK2 on Tyr^1022^-Tyr^1023^ and Tyr^1054^-Tyr^1055^ residues, respectively. (3) Activated TYK2 can then phosphorylate the serine/threonine kinase PDK2 which in turn (4) phosphorylates the two serine residues of the IFNAR1 phosphodegron ^534^D**S**GNY**S** ^539^. It acts as a docking site (5) for the Skp1-Cullin1-F-box complex E3 ubiquitin ligase (SCF^βTrcp^). (6) Once bound to IFNAR1, SCF^βTrcp^ is able to polyubiquitinate (blue spheres) lysine residues 501, 525 and 526 by adding Lys^48^ and Lys^63^ linkages. In parallel, the endocytic linear motif ^466^YVFF^469^ recruits AP50, the μ2 subunit of AP2 adaptor complex. (7) Together, AP50 binding and IFNAR1 polyubiquitination trigger the association of IFNAR receptor complex with the clathrin machinery and its endocytosis *via* clathrin-coated vesicles.

The lack of IFNAR intrinsic tyrosine kinase activity is compensated by a non-covalent and constitutive association with Janus tyrosine Kinases (JAK). The JAK family is composed of four members: JAK1 and JAK2 (22), TYK2 (23, 24), and JAK3 (25). JAKs are large multidomain proteins with an N-terminal part dedicated to the recognition and interaction with the PM proximal region of the receptor and a C-terminal part regulating its kinase activity (**Figures 1A, B**) [for more details see reviews (26, 27)]. IFNAR1 is constitutively associated with TYK2 (28, 29) and IFNAR2 with JAK1 (13, 30, 31). IFN-induced association of the two IFNAR chains allow JAK1 and TYK2 to form a functional signaling unit (**Figure 1C**). The precise mapping of interactions between IFNAR and associated JAK are well described (32, 33). TYK2 interaction with IFNAR1 was shown to stabilize IFNAR1 at the PM (34–36) thereby controlling IFNAR1 PM pool. Moreover, both JAK1 and TYK2 play a central role in IFNAR trafficking and signaling by regulating the recruitment and/or the activity of other IFNAR interactors through JAK-dependent tyrosine phosphorylation.

The canonical type I IFN signaling pathway relies on the phosphorylation and nuclear translocation of signal transducers and activators of transcription (STAT) proteins. STATs share a similar structure with an SH2 domain that allows the interaction with cytokine receptors and regulators, and a DNA binding domain that modulates gene transcription (37). Pull-down experiments suggested that STAT2 was constitutively associated with the cytosolic domain of IFNAR2 (**Figure 1B**) (38). This interaction was further confirmed and visualized in cells expressing HaloTag-IFNAR2 spatially constrained at the PM by HaloTag ligand functionalized on a micropatterned surface (39). Whereas STAT2-EGFP showed a strong colocalization with patterned IFNAR2, a very limited colocalization of STAT1-EGFP could be detected in the same conditions. This probably reflects that STAT1 docking to IFNAR2 occurs through STAT1-STAT2 heterodimerization in agreement with the requirement of STAT2 for STAT1 phosphorylation by IFN (37, 40). These results suggest that additional sites for STAT docking may be created by IFNAR phosphorylation, thus enhancing binding of STAT1 and STAT2. The precise mapping of STAT2 interaction that is still unknown should lead to a better understanding of the IFNAR complex organization.

STAT2 mediates the recruitment of the ubiquitin specific peptidase 18 (USP18) to the IFNAR2 subunit (**Figure 1B**). USP18 is an isopeptidase that promotes the de-conjugation of the ubiquitin-like modifier interferon-stimulated gene 15 (ISG15) (41), a reaction known as deISGylation. USP18 is first recruited to STAT2 (42), and then to IFNAR2 where it competes with JAK1 binding, which results in JAK/STAT signaling inhibition (43). USP18 binding to IFNAR2 stabilizes the interaction of STAT2 with IFNAR2 (39) and inhibits IFN-α binding (44, 45). Thus, both STAT2 and USP18 are important for IFNAR complex trafficking and signaling.

The adaptor protein receptor for activated protein kinase C-1 (RACK-1) is also constitutively associated with IFNAR2 (46). This interaction is maintained after IFN stimulation. RACK-1 is a scaffold protein with no enzymatic activity, which recruits specific signaling elements. RACK-1 directly interacts with IFNAR2, TYK2, and JAK1, and indirectly with IFNAR1 (47). RACK1 is necessary for STAT1 and STAT2 phosphorylation. The RACK-1 binding site on IFNAR2, partially overlaps with JAK1 binding site.

### Type I Interferon Receptor Proper Function Depends on Posttranslational Modifications

Receptor glycosylation is an important posttranslational modification regulating their activity through various mechanisms including proper folding in the ER, localization at the PM (*via* interactions with galectins and with gangliosides in microdomains for instance), ligand binding, intracellular trafficking and signaling (48, 49). The two IFNAR subunit extracellular domains are highly glycosylated resulting in their high molecular weight (MW) (**Figures 1A, B**). Thus, human IFNAR1 has an approximately 130 kDa MW instead of the theoretically calculated 63.5 kDa MW corresponding to the 557 amino acids (aa) of IFNAR1, and human IFNAR2 has a 100-110 kDa MW instead of a theoretical 57.8 kDa MW for its 515 aa (50).

Levels of IFNAR1 and IFNAR2 at PM are likely to be regulated independently from each other. The PEPD prolidase is required for the maturation and surface expression of IFNAR1 but not of IFNAR2 (51). The importance of these posttranslational modifications in IFN biological activity is illustrated by NS5 viral proteins that are produced by some Flaviviruses. NS5 can suppress IFNAR1 glycosylation by binding to PEPD thereby interfering with IFNAR1 delivery from the trans-Golgi network (TGN) to the PM (51, 52). Hence IFNAR1 glycosylation is likely to play an important role in signaling by regulating IFNAR1 density at the cell surface.

Both IFNAR chains are palmitoylated on cysteins (**Figures 1A, B**) (53). Palmitoylation has been involved in the proper addressing of proteins from the TGN to the PM, in protein-protein interactions, and in the association with lipid nanodomains, all steps that are important for the targeting, stability and function of receptors at the PM (54). IFNAR1 is mono-palmitoylated on Cys^463^ and the corresponding C463A IFNAR1 mutant, totally lacking palmitoylation, showed no alteration either in stability at the PM, IFN-α-induced endocytosis or intracellular distribution but showed some defects in the later steps of JAK/STAT signaling (53). Indeed, although the very first events of JAK/STAT signaling (i.e. JAK1, TYK2 and IFNAR1 tyrosine phosphorylations) were unchanged, IFNAR1 palmitoylation was found to be required for STAT1 and STAT2 downstream phosphorylation and nuclear translocation. Therefore, IFNAR1 palmitoylation may participate to target IFNAR1 to the proper PM nanodomains or the recruitment of effectors associated to the signaling receptor complex. Although IFNAR palmitoylation is not involved in IFNAR1 addressing to the PM and IFNAR1 internalization, it is worth noting that the inhibition of global protein palmitoylation by 2-bromopalmitate strongly affects IFNAR1 internalization after IFN stimulation, suggesting that yet to be identified palmitoylated effectors, associated or not with the IFNAR complex control these processes. Although little is known about the molecular mechanisms linking palmitoylation and JAK/STAT signaling, this posttranslational modification remains an interesting avenue of investigation, as it may help us to reveal new links between IFNAR subunit trafficking and its signaling.

Whether multi-chain cytokine receptors are pre-associated or not before their activation at the PM remains highly debated for some members of this family as recently illustrated by the IFN gamma receptor (55, 56). The debate seems closed for IFNAR as several studies convincingly established that IFNAR1 and IFNAR2 are not pre-associated at steady state and that receptor dimerization occurs only upon ligand binding (57–60). The two chains are nevertheless associated to other partners such as lipids or proteins, whose main function is to maintain the receptor at the PM and keep the receptor-bound JAK kinases inactive.

### Receptor Partitioning in Plasma Membrane Nanodomains

Based on IFNAR density at the cell surface, the constant rate of ligand association/dissociation predicts that IFNAR1 and IFNAR2 subunits should not be able to form stable complexes if they were uniformly distributed (59). The two chains of IFNAR should therefore be clustered in the same local structures at the PM. Twenty years ago, in mouse embryonic fibroblasts (60), IFNAR1, like many other receptors at that time (61), was proposed to be associated with lipid rafts, these PM asymmetric lipid assemblies thought to be detected in detergent resistant membranes (DRM). If this technique is today outdated, more sophisticated cell imaging methods have led to a finest characterization of the nanoscale distribution of IFNAR at the PM.

Caveolae are characteristic small buds present at the PM of many cell types, that are enriched in cholesterol and glycosphingolipids, and coated with the structural protein caveolins (Cav) and assembly proteins cavins (62). The early finding that caveolae could biochemically fractionate in DRM fractions has led to refer to them as a subtype of lipid rafts (63). While this definition is not accurate any longer (64), the role of caveolae in IFNAR distribution has remained elusive. Indeed, early electron microscopy (EM) studies failed to detect human IFN-α or murine IFN-β in caveolae (65, 66). More recently, the overexpression of the chain cytokine receptor family B1 (CRFB1), the zebrafish IFNAR ortholog, resulted in its colocalization with Cav1β domains at the PM using super resolution microscopy (67). The finding that Cav1β depletion significantly decreased CRFB1 PM clustering and IFN-induced STAT1 signaling is in strong support of an important role of Cav1β in zebrafish IFN-R system and not of caveolae since the other caveolae constituents like cavins were not studied. However, the analysis of gene sequences and structures of zebrafish IFN-R complexes such as CRFB5 chain associated with either CRFB1 or CRFB2, revealed important differences with mammalian IFNAR and closer homology with IFN-λR (68, 69). IFN-φ was proposed as a new nomenclature for fish IFN to close the debate (70). The regulation of CRFB1 by Cav1β seems therefore less relevant to human IFNAR for which no strong evidence of a role of caveolae and/or caveolin was brought so far.

By using super-resolution microscopy restricted to the PM, a study revealed that overexpressed IFNAR1 and IFNAR2 were partially co-clustered in nanoscale domains (71). These clusters were weakly co-localized with clathrin, the structural core protein of endocytic clathrin-coated pits, but frequently found in the vicinity of actin structures. On the contrary, a new study by the same team based on quantitative ligand-binding with fluorescently-labeled IFNs described the presence of continuously diffusing IFNAR in a random and non-clustered distribution at the PM (20). This apparent discrepancy could result from the high density of receptors caused by their overexpression, which could artificially induce IFNAR1/IFNAR2 co-clustering at steady state (72). Nevertheless, the confinement of the receptor chains at physiological density (5–10 chains per μm²) in the cortical actin meshwork was confirmed by single quantum dot tracking and localization microscopy (73). In this study, the longer lifetime of the IFN-α2 induced ternary complex measured in cells in comparison with artificial membranes *in vitro* was attributed to its stabilization through an active nano-confinement in 70 nm cortical actin sub-domains organizing larger domains of 300 nm. Disrupting these domains decreased the stability of the ternary complex and JAK/STAT downstream signaling. In summary, these studies indicate that IFNAR chains are confined in actin-dependent nanodomains at the PM. It is likely that the continuous technological developments in super-resolution microscopy will reveal new features of IFNAR nanoscale partitioning and functions at the PM that could not be detected with conventional fluorescence microscopy.

## The Basic Interferon—Type I Interferon Receptor Complex

Binding of IFN to IFNAR2 is followed by IFNAR1 association with the IFN/IFNAR2 subcomplex (**Figure 1C**). IFNAR1 binding is associated with a major conformational change involving movement within the four extracellular SD domains, resulting in the efficient capping of IFN molecule (74, 75). IFN binding has also been shown to reduce the force needed to unfold the IFNAR1 extracellular domains (76). The reduction in IFNAR1 rigidity would enable the propagation of IFN-induced conformational changes closer to or even across the PM. Interestingly, comparison of the ternary complexes formed by the two IFNAR1 and IFNAR2 receptor chains with IFN-α or IFN-β by single-particle EM analysis could not reveal any difference (74). Likewise, no difference could be detected regarding the IFNAR1 residues involved in the interaction with IFN-α or IFN-β. For instance, IFN-β maintains the same overall fold, and shares the same binding interface with IFNAR1 and IFNAR2, similarly to IFN-α2 (10). Interestingly, it was observed that among the IFN subtypes, increased binding affinities are correlated with a higher rate of IFN-IFNAR endocytosis (75). This faster entry within the endosomal system has been proposed to lead to earlier signal triggering but also to rapid termination. Thus, it was proposed that it could explain, at least partially, some of the signaling differences, such as anti-proliferative activities, observed between IFN-α, IFN-β, and IFN-ω.

Upon formation of the ternary complex, JAK1 and TYK2 are both brought in close contact, which leads to a repositioning of their respective pseudokinase domain, thereby relieving the self-inhibition by the JH1/JH2 domains (77). While the exact underlying molecular mechanism remains debated, the first activating events would involve the concomitant tyrosine phosphorylation of JAK1 and TYK2 on Tyr^1022^-Tyr^1023^ and Tyr^1054^-Tyr^1055^ residues, respectively (**Figure 1C**) (78).

Following JAK activation, several other post-translational modifications within the receptor complex lead to the recruitment and regulation of new partners, to the internalization of the complex and also to the priming of the IFN-induced signaling cascade. Several of these steps have been studied but there is still a lot of mechanisms of this finely tuned pathway that need further investigation.

Upon IFN stimulation, the serine/threonine kinase PKD2 is recruited to IFNAR1. PKD2 is then TYK2 phosphorylated on Tyr^438^ (79) and activated PKD2 can in turn phosphorylate IFNAR1 on Ser^535^ and Ser^539^ residues within the ^534^DSGNYS^539^ phosphodegron—also called destruction motif (**Figure 1C**) (80). The SCF^βTrcp^ (Skp1-Cullin1-F-box complex) E3 ubiquitin ligase binds to the destruction motif of IFNAR1 and adds polyubiquitin chains on lysine residues 501, 525, and 526 (80, 81). The ubiquitination process plays an import role in the internalization of the IFNAR complex since interfering with either Ser^535^ phosphorylation, SCF^βTrcp^ recruitment or polyubiquitination inhibits endocytosis (82). TYK2 kinase activity is essential for IFNAR1 Ser^535^ phosphorylation, which in turn is required for IFNAR1 ubiquitination-dependent endocytosis. It is therefore expected that IFNAR1 Ser^535^ phosphorylation and ubiquitination should represent the very first steps that follow the reunion of the two chains of the receptor initiated by IFN binding.

## Beginning of the Journey: En Route to the Endosome

**Figure 2:**
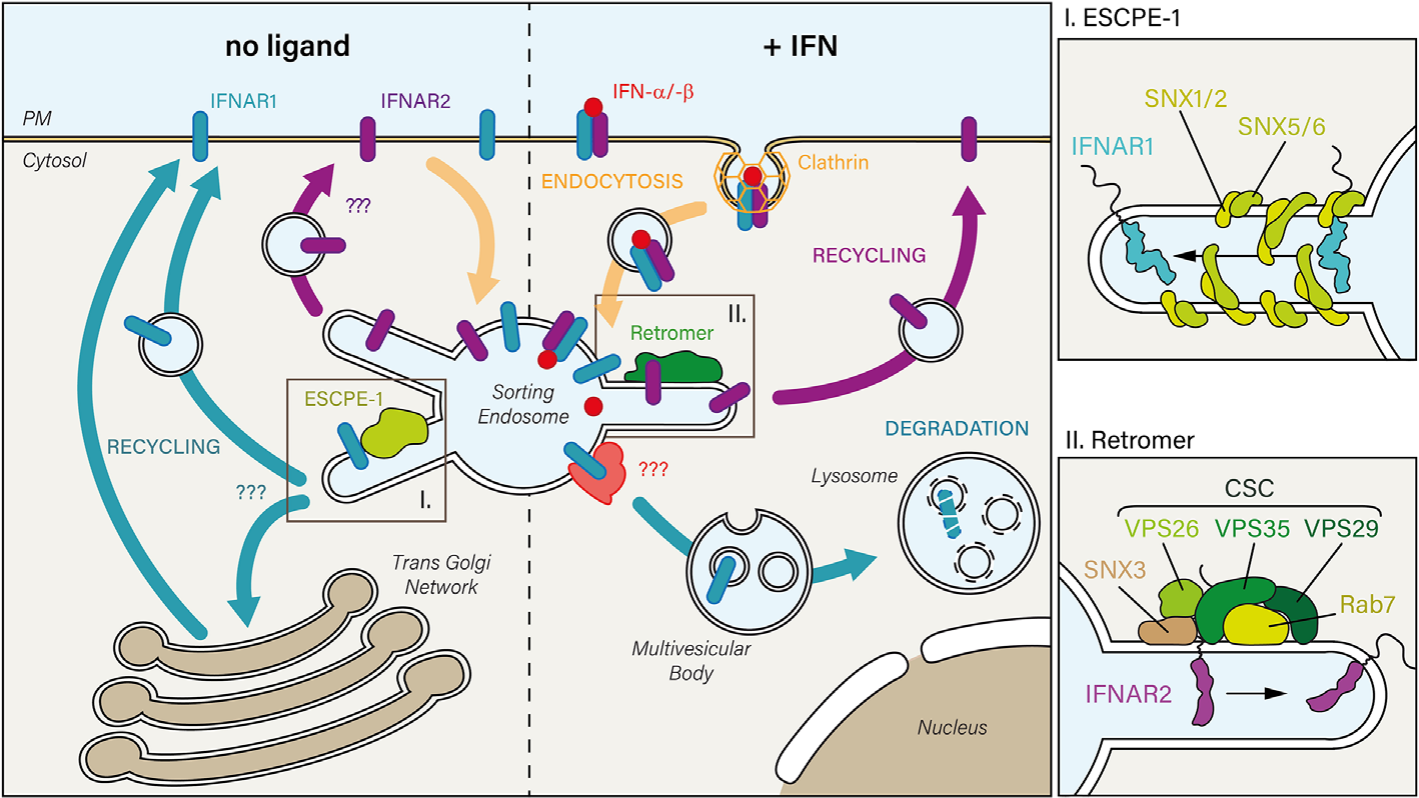
In the absence of IFN (left), constitutively internalized IFNAR1 and IFNAR2 subunits may recycle from the sorting endosome to the PM. While the mechanism is unknown for IFNAR2, IFNAR1 is sorted back to the PM through its interaction with the endosomal sorting nexin (SNX)-BAR sorting complex for promoting exit 1 (ESCPE-1). ESCPE-1 complex (top right inset) is composed of SNX heterodimers formed by SNX1 or SNX2 that are associated with SNX5 or SNX6. Whether IFNAR1 transits through the trans-Golgi Network during its recycling to the PM has not been investigated yet. Upon IFN stimulation (right), the IFN-bound receptor complex is endocytosed *via* clathrin-coated pits. Upon arrival to the sorting endosome, IFNAR receptor complex is dissociated: 1) IFNAR1 is addressed to the lysosomal degradative pathway, first through multivesicular bodies, then in lysosomes where it is fully degraded, and 2) IFNAR2 interacts with the endosomal retromer complex which controls its recycling to the PM. The retromer (bottom right inset) is formed by the cargo-selective-complex (CSC), a trimer composed of vacuolar protein sorting-associated protein 35 (VPS35)-VPS29-VPS26 proteins, associated with the nexin SNX3 and the small GTPase Rab 7.

IFNAR, like most transmembrane signaling receptors, is endocytosed by clathrin mediated endocytosis (CME). Earlier EM studies localized IFN-α (65) and IFN-β (66) in clathrin coated pits (CCP). The role of CME in IFNAR uptake was definitely established by the finding that IFNAR uptake required key elements of the clathrin-dependent endocytosis machinery (**Figure 2**) including clathrin heavy chain, the α2 adaptin protein-2 (AP2) complex, the GTPase dynamin and Eps15 (53, 82, 83).

CME is initiated at the PM by the recruitment of transmembrane receptors to the clathrin machinery thanks to the interaction of the AP2 adaptor complex with a tyrosine-based linear endocytic motif YXXΦ that is found in the receptor cytoplasmic tail (7). Although this endocytic motif is present in many transmembrane receptors, very few examples have documented a direct interaction with AP2 (5, 6, 84). It is therefore worth noting that AP50, the μ2 subunit of AP2, was shown to recognize ^466^YVFF^469^ domain in IFNAR1 (**Figure 1A**), a canonical tyrosine-based linear endocytic motif. At steady state, the interaction between IFNAR1 and AP50 is prevented by the physical masking of ^466^YVFF^469^ by TYK2 (36). The endocytic motif is in close proximity to the TYK2 minimal interaction domain (486-511) and is inserted in the TYK2 maximal interaction domain (465-511) (32). It was shown that IFNAR1 stability at the PM is reduced in the absence of TYK2, probably by allowing at steady state the interaction of the otherwise masked endocytic motif with AP50 and thus the internalization of IFNAR1 (34–36). Upon ligand binding, one can speculate that the endocytic motif would be unmasked by TYK2 as a result of the various conformational changes associated with IFNAR chains complexes and JAK rearrangement and activation.

The ^466^YVFF^469^ motif is highly conserved within species except for the mouse (36), which could explain why the stability of murine IFNAR1 at the PM was not affected by the absence of TYK2 (85–87). This does not explain however the mechanism by which the AP2 complex would be recruited to the murine receptor. Other sequences such as di-leucine and iso-leucine can also be recognized by clathrin adaptors subunits (84). Indeed, leucine-based motifs are found in the mouse but also in the human IFNAR1. While it is likely that these sequences can also modulate the uptake of IFNAR, no study has so far documented their function. It is also possible that the endocytosis of murine IFNAR depends exclusively on IFNAR1 ubiquitination since the S526A ubiquitination deficient mutant, which is unable to interact with SCF^βTrcp^, shows a significant reduced internalization (80). Finally, one cannot rule out a participation of several of these mechanisms in a process as finely tuned as CME.

If the importance of IFNAR1 Tyr^466^ in clathrin-dependent endocytosis is clear, the role of phosphorylated Tyr^466^ is more obscure. Phosphorylation of the tyrosine motif YXXΦ is likely to inhibit AP50 recruitment, as the negatively charged phosphate group would prevent the endocytic motif to fit into AP50 binding pocket (88, 89). The fact that IFNAR1 Tyr^466^ phosphorylation occurs as early as 5 min after IFN-α stimulation (29) questions the chronology of these events within the time range of internalization and signaling. That AP50 cannot interact with the phosphorylated (pY)XXΦ motif (90) infers that IFNAR1 Tyr^466^ should not be phosphorylated during the recruitment of the endocytic machinery. It was suggested that Tyr^466^ was phosphorylated immediately after ligand binding, and then dephosphorylated by the PTP1B phosphatase, thereby allowing the interaction with AP50 (91). This phosphorylation/dephosphorylation cycle is, however, unlikely if one considers that IFNAR1 is endocytosed very rapidly after IFN stimulation whereas IFNAR1 Tyr^466^ phosphorylation can still be detected by western blotting at longer time points (92). Although experimental evidence is yet lacking, it seems more likely that IFNAR1 Tyr^466^ phosphorylation occurs after AP50 binding. Whether it happens at the PM just after AP50 recruitment to the IFNAR1 or later after IFNAR complex endocytosis is unknown. In this context, it was shown that AP50 binding to the EGFR, that occurs only when the YXXΦ motif is not phosphorylated, was still maintained after phosphorylation of the YXXΦ motif (90).

The formation of the IFNAR complex allows the activation and recruitment of additional regulating partners such as phosphatases (SHP1, SHP2, PTP1B, TCPTP, CD45) or SH2 domain-containing proteins (SOCS, LNK) [reviewed in (77, 78)]. Whether the recruitment of these effectors takes place at the PM or later along the endocytic route is a question that is difficult to tackle because of the intrinsic rapidity of the endocytic process. New approaches such as the functionalization of micropatterned surface with Halo ligands that retain IFN-IFNAR complex at the PM will help to better understand these processes (39).

During acute brain infection by the simian immunodeficiency virus (SIV), IFN-α signaling is drastically hampered with a down-regulation of downstream effectors such as TYK2, STAT1 and IRF7, in contrast to IFN-β signaling that remains fully active (93). A follow-up study using SIV as a model of infection in macrophages, revealed that the inhibition of IFN-α signaling in the central nervous system was triggered by CCL2, a chemokine secreted by astrocytes (94). Interestingly, confocal microscopy revealed in leukocytes that β-arrestin 2 was recruited to endomembranes positive for CCR2B, the CCL2 receptor, upon CCL2 treatment (95). β-arrestins (1 and 2) play a central role in GPCR desensitization, internalization and intracellular trafficking. They act as adapters that build a bridge between activated i.e. phosphorylated GPCRs and the two main components of the clathrin-dependent endocytic machinery, AP-2, and clathrin (96–98). In support of this, it was recently reported that silencing RNA against β-arrestin 2, but not β-arrestin 1, restored IFN-α levels of SIV infected macrophages in a CCL2/CCR2-dependent manner (99). They further showed that β-arrestin2 was required for IFNAR1 internalization, in agreement with the first implication of AP2 in IFNAR1 endocytosis (83).

### Type I Interferon Receptor Endocytosis Is Mandatory for JAK/STAT Signaling

**Figure 3:**
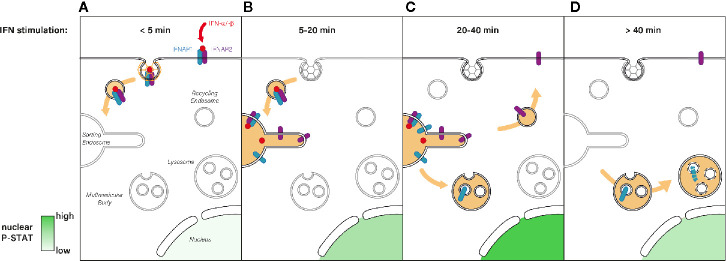
**(A)** Immediately after type I IFN binding the IFNAR1-IFNAR2 receptor complex is actively endocytosed from the PM and addressed to the endosomal network. **(B)** Five to 20 min after IFN stimulation, the IFN-IFNAR complex arrives at the sorting endosome where JAK/STAT signaling is triggered, which results in P-STAT nuclear translocation. **(C)** For the following 20 min, IFNAR signaling is maintained at the endosome where it reaches its maximum (highest P-STAT1 level). The subsequent sorting of IFNAR1 to multivesicular bodies results in signaling termination whereas IFNAR2 is recycled to the PM. **(D)** After 40 min of IFN stimulation, IFNAR1 is degraded through the lysosomal pathway whereas IFNAR2 is ready for a new round of IFN binding at the PM. Nuclear levels of P-STAT return to pre-stimulation levels.

Although JAK/STAT signaling has a pivotal role in key cellular processes, the underlying molecular mechanisms controlling its activation by IFNAR and the determination of IFN signal specificity have remained poorly understood (100–104). In 2006, in line with the original studies establishing the role of endocytosis in the regulation of receptor signaling, the Lamaze group revealed for the first time that IFNAR endocytosis was required to trigger JAK/STAT signaling downstream of IFN-α stimulation (83) (**Figure 3**). Blocking IFNAR CME with the dominant negative mutant DynK44A or a siRNA against clathrin heavy chain strongly decreased the level of STAT1 and STAT2 phosphorylation induced by IFN-α but not by IFN-γ. Accordingly, it was later shown that IFN-γ receptor subunits need to be associated with specific cholesterol/sphingolipid enriched PM nanodomains for JAK/STAT activation IFN-γ, independently from receptor endocytosis (55). The essential role of CME in JAK/STAT signaling was further confirmed a year later in Drosophila with the receptor Domeless (Dome) whose internalization is also required to transduce JAK/STAT signaling, (105). Like IFNAR1 and IFNAR2, Dome presents fibronectin type-III extracellular domains (106) and a conserved di-leucine motif in its cytosolic tail (107). These data emphasize that the control of JAK/STAT signaling by receptor endocytosis is a conserved mechanism among species.

## Sorting Endosome: The Place to Be

Today, the central role of the endosomal network in membrane trafficking is not questioned. Endosomes have been classically divided into early and late endosomes to reflect the chronology of cargo delivery. Early endosomes represent the first intracellular station downstream of endocytosis at the PM, where receptors are sorted from ligands. The central function of early endosomes in cargo sorting led to rename them as sorting endosomes [for review see (108, 109)]. In the late 1990s, two seminal studies simultaneously revealed that the sorting endosome could serve as a relay where receptor signaling could be controlled. Thus, nerve growth factor (NGF) and its receptor gp140^TrkA^ (TrkA) were rapidly endocytosed by CME into early endosomes where TrkA -bound NGF could activate TrkA resulting in TrkA tyrosine phosphorylation and in the binding to the downstream effector PLC-γ1 (110). At the same time, another study took advantage of the first molecular possibility to selectively inhibit clathrin-dependent endocytosis using a dominant negative mutant of dynamin, the GTPase required for the scission of clathrin-coated pits from the PM (111). Inhibition of EGF receptor (EGFR) endocytosis with the dynamin mutant revealed that in addition to attenuate EGFR signaling, endocytosis was also required to deliver activated EGFR in early endosomes where EGF specific signaling pathways could be either activated or terminated (112). These pioneering studies established the essential role that endosomes could play in the control of signal transduction. This groundbreaking work has been followed by numerous studies that contributed to definitely upgrade the early endosome from a sorting organelle to an active signaling hub and to establish the new concept of the *signaling endosome*. The signaling endosome has since been extensively investigated and became a major center of interest in the understanding of the cell biology of signaling receptor trafficking and signaling as described in many reviews (113–118).

The requirement for IFNAR endocytosis in JAK/STAT signaling implies that endosomal sorting machineries should be at work in this process. Recent studies have indeed uncovered the role of a major endosomal sorting machineries in IFNAR trafficking and signaling, namely the endosomal sorting complex required for transport (ESCRT). ESCRT has a central position as the major complex mediating the entry of ubiquitinated cargos into the lysosomal degradation pathway (119, 120). The ESCRT machinery consists of four protein complexes: ESCRT-0, -I, -II, and -III, and include several accessory components that are highly conserved from yeast to human. Over the past decade, structural and biochemical studies have uncovered the sequential process by which ESCRT assembly occurs [reviewed in (121–123)]. ESCRT-0 is tethered on the endosomal membrane where it recruits ubiquitinated cargos and interacts with ESCRT-I. ESCRT-0 is composed of two subunits: hepatocyte growth factor-regulated tyrosine kinase substrate (Hrs), which binds to the ubiquitinated substrate, and signal-transducing adaptor molecule (STAM) (124–127). Hrs and its partner STAM, both ubiquitously expressed, play a vital role in biology and development as shown by the death of Hrs deficient mice *in utero* (128–130). Hrs is recruited to the early endosome where it binds to endosomal specific PI(3)P phosphoinositide through its FYVE-domain and initiates the formation of intraluminal vesicles (ILV) found inside multivesicular bodies (MVB) (123).

During the last decades, our understanding of the cellular functions of ESCRT have evolved from a sequential process controlling cargo entry into the lysosomal degradation pathway to more diverse biological activities such as virus budding, membrane repair, cytokinesis, regulation of gene transcription, autophagy, quality control of nuclear pore complexes (131–137).

### Hrs/STAM for Type I Interferon Receptor Sorting: Insights From *Drosophila*

Following the first report of EGFR signaling control by clathrin-dependent endocytosis (112), efforts were directed at elucidating the molecular mechanisms underpinning EGFR endosomal sorting and signaling. Thus, EGFR sorting toward the lysosomal degradation pathway was found to require Hrs recognition of EGF-induced ubiquitinated EGFR (113, 138–142). Before arrival to the lysosome, ubiquitinated EGFR accumulate into ILVs of MVBs (143). This physically removes the signaling tail of EGFR from the cytosol, effectively terminating the downstream signaling cascade (144). In this context, cells depleted for the *Hrs* gene revealed that Hrs negatively controls EGFR signaling in Drosophila embryogenesis with an increase of EGFR signaling activity and an accumulation of ubiquitinated cargos in enlarged endosomes, including EGFR and other activated signaling receptors such as PDGF/VEGF receptors, or Notch (105, 145–147).

Based on the prototypical example of EGFR sorting and signaling control by Hrs, numerous studies have addressed the role of Hrs in receptor signaling especially for GPCRs. Thus, the β_2_-adrenergic receptor (β_2_AR) and the δ-opioid neuropeptide receptor (DOR), are two examples of GPCRs that are regulated by Hrs. Both receptors are ubiquitinated on lysine residues in the carboxyl terminal tail and are endocytosed in a clathrin- and β-arrestin-dependent manner (148–155). Similarly to EGFR, DOR sorting toward the lysosomal degradation pathway requires Hrs (156). Hrs has a different effect on β_2_AR since it mediates its recycling to the PM, a process associated with β_2_AR resensitization (157). Hrs-dependent recycling was shown to rely on an acidic di-leucine motif present in the C-terminal tail of the β_2_AR but not on ubiquitination (158, 159). Hrs and STAM were also required for efficient fibroblast growth factor receptor (FGFR) endosomal sorting and signaling in Drosophila. This study revealed an opposite role of Hrs and STAM depending on the location of the complex since EGFR signaling was downregulated by Hrs and STAM in the embryo but fully activated during wing development (147).

The role of Hrs in IFNAR trafficking and signaling had not been investigated until recently. A first evidence came from studies in Drosophila where the disruption of Hrs in egg chambers transiently expressing Dome receptor led to an inhibition of STAT activation upon Dome stimulation (105). A recent study showed the constitutive association of STAM2, without Hrs, with IFNAR1 and TYK2 at the PM preventing TYK2 activation by IFN (160). IFN-α induced receptor endocytosis delivers the STAM-IFNAR1-TYK2 complex to early endosomes positive for PI(3)P where Hrs interaction abolishes STAM inhibitory effect and triggers IFNAR endosomal signaling. In contrast, IFN-β stimulation results in IFNAR sorting to a distinct endosomal subdomain where endosomal JAK/STAT signaling occurs independently from Hrs. This study put into question the classical dogma stating that Hrs and STAM are always constitutively associated on the endosomal membrane to form the ESCRT-0 complex. Indeed, a few studies suggest that this assumption may suffer some exceptions. Thus, a truncated mutant of STAM lacking the coiled-coil domain of interaction with Hrs was reported to promote the relocation of Hrs in the cytoplasm (161). Hrs was reported to be targeted to early endosomes independently of STAM, and STAM was also localized at the PM in HeLa cells (162). In line with this study, a recombinant Hrs purified in the absence of STAM could be detected on membranes as hexamers (163).

The role of Hrs/STAM in cytokine signaling is not exclusive to JAK/STAT signaling by the IFNAR complex. Indeed, more than fifty cytokines can signal *via* the JAK/STAT pathway (164). Among them, IL-4 signals through both the type I receptor consisting of the IL-4Rα and the common gamma chain (γC), and the type II receptor composed of IL-4Rα and IL-13Rα1 (164). IL-2 activates JAK/STAT signaling downstream of the trimeric IL-2R composed of α, β, and γC chains (165). Hrs controls IL-2R and IL-4R signaling albeit through its classical regulatory function as it is required for IL-4Rα and IL-2Rβ endosomal sorting toward the degradation pathway, resulting in receptor cell surface downregulation and signaling termination. Thus, in contrast to IFNAR1, the binding of Hrs on IL-4Rα and IL-2Rβ is not required for endosomal signaling, (166, 167). Finally, as reported above for GPCRs, Hrs can regulate IL-2Rα recycling to the PM by binding to a hydrophobic amino acid cluster in an ubiquitin-independent manner (167). The absence of such motifs in the C-terminal tail of IFNAR1 and IFNAR2 may explain why Hrs is not involved in IFNAR recycling (see below).

## Endosomal Exit: Choose Your Destiny

After internalization and arrival in early endosomes, cargos are classically sorted to three possible destinations: (1) fast recycling to the cell surface *via* the endosomal recycling pathway, (2) recycling to the PM through the retromer or retriever complexes or *via* the TGN (retrograde recycling), and (3) degradation in lysosomes. Although these three main routes have been investigated for several signaling membrane receptors, mainly EGFR, IL2-R, and growth hormone receptor (GHR), the intracellular fate of the IFNAR receptor complex upon its endocytosis has long remained mysterious.

### Type I Interferon Receptor Recycling to the Plasma Membrane

In contrast to IFNAR1, whose final fate is degradation in the lysosome (see below), IFNAR2, which is not ubiquitinated, takes a different path. IFNAR2 intracellular trafficking has long remained poorly characterized. A first study measured unchanged IFNAR2 levels at the PM during IFN-α stimulation while IFNAR1 was efficiently degraded (168). These data suggested that after IFNAR endocytosis, IFNAR2 was probably recycled back to the PM by unknown mechanisms.

The retromer complex plays a central role in both the retrograde transport of endosomal proteins to the TGN and in the endosomal recycling of cargos to the PM (169–171). The retromer complex is assembled by a first sub-complex called cargo-selective-complex (CSC), a trimer made of vacuolar protein sorting-associated protein 35 (VPS35)-VPS29-VPS26 proteins, that are highly conserved among species (169, 172, 173). The CSC binds PI(3)P, an early endosome specific phosphoinositide, through the Phox-homology (PX) domain present in sorting nexins (SNX), which together with the small GTPase Rab7a, assembles the second sub-complex of the retromer (174, 175). In addition to membrane tethering, SNX bend and remodel the endosomal membrane to create recycling tubules for cargo trafficking [reviewed in (176)]. Cargo selection is mediated through the FERM-like domain of SNX17 whereas SNX27 interacts with cargos *via* its PDZ domain. Recently, SNX17 has been shown to interact with another endosomal sorting complex called the retriever, a heterotrimer harboring similarities with the retromer. The retriever and SNX17 can associate with other complexes to prevent cargo lysosomal degradation and to promote cell surface recycling (177).

Upon IFN-α stimulation, IFNAR2, in contrast to IFNAR1, could not be colocalized with LAMP1, a *bona fide* marker of late endosomes and lysosomes (21). Instead, IFNAR2 was accumulated in early endosomes in the absence of Rab11A or Rab4, two GTPases involved in cargo recycling to the PM. These findings therefore suggest that IFNAR2 is not directed towards the lysosomal degradation pathway but recycled back to the PM. Indeed, mass spectrometry analysis revealed that upon IFN-α stimulation, IFNAR2 could interact with Rab11A and Rab4. IFNAR2 could also interact with Rab35 and VPS26A, VPS29 and VPS35 – the components of the retromer cargo-recognition trimer (**Figure 2**). Accordingly, under IFN stimulation, cells depleted of VPS35 accumulated IFNAR2 in early endosomes together with a decreased amount of IFNAR1 sorted for lysosomal degradation. The same phenotype was observed in the absence of IFN stimulation, indicating that IFNAR is sorted by the retromer complex under basal and stimulated conditions. In agreement with the interaction of the Rab7 GTPase with the retromer complex (175), Rab7 depletion led to the same phenotype than Vps35 depletion. These data agree with a previous study showing an accumulation of IFNAR1 in early endosomes in Rab7A depleted cells (178). Although the retromer complex has been involved in the retrograde trafficking of mannose-6-phosphate (179), sortilin-related (180) and Wnt receptors (181) from endosomes to the TGN, the possibility of IFNAR2 retrograde trafficking to the TGN by the retromer complex was ruled out (21).

A recent proteomic study identified IFNAR1 as a possible cargo recognized by SNX5 nexin through binding to ^466^YVFFP^470^, a consensus ФxΩxФ recycling motif (182). Interestingly, the SNX5-binding motif covers the endocytic motif ^466^YVFF^469^ described before (36). The endosomal SNX-BAR sorting complex for promoting exit 1 (ESCPE-1), identified in this study, allows to couple cargo recognition with SNX-mediated biogenesis of tubulo-vesicular transport carriers that recycle cargo to the PM or send them to the TGN (182). Whether this new recycling pathway mediates IFNAR1 basal recycling to the PM will need further investigation (**Figure 2**).

### Degradation of Type I Interferon Receptor 1 in Lysosomes

Early studies performed with human radiolabelled ^125^I-IFN-α led to the first report of the degradation of IFN-α after IFNAR endocytosis, probably in lysosomes since it was blocked by chloroquine, a lysosomotropic agent (65, 183). IFNAR1 degradation was first described after IFN-α stimulation of Daudi cells (19). The lysosomal proteolysis of IFNAR1, excluding proteosomal degradation, was later established with lysosome inhibitors (80, 184). Several IFN-induced post-translational modifications in the cytosolic tail of IFNAR1 are necessary for its targeting to lysosomes. Upon IFN stimulation, the ubiquitination of IFNAR1 proximal lysine residues adds K48 and K63 linkages which are known to sort polyubiquitinated cargos to the proteasome or lysosomes for degradation (**Figure 2**) (81, 185, 186).

IFNAR1 ubiquitination occurs probably mainly at the PM after IFN binding, but could also take place at the endosomal level as described for GHR (187). This study showed that SCF^β-Trcp^ was active at the cell surface and in endosomes. Silencing SCF^β-Trcp^ or deleting the GHR ubiquitin-dependent endocytosis motif forced GHR recycling from endosomes to the PM, indicating that GHR sorting to lysosomes depends on an active ubiquitin system. More recently, several studies revealed that ubiquitinated receptors can be sorted to ILVs independently from ESCRT as shown for GPCR with ALG-2-interacting Protein X (ALIX) or EGFR, PDGFR and α5β1 integrin with the histidine domain phosphotyrosine phosphatase (HD-PTP) [review in (188)]. The precise molecular mechanism mediating IFNAR1 lysosomal degradation will require further investigations. Whether ALIX and HD-PTP are part of IFNAR1 sorting machinery are still open questions.

### Type I Interferon Receptor Complex Dissociation and Signaling Termination

Whether and how the retromer complex contributes to the regulation of intracellular signaling are still poorly understood. How ligand-induced receptor phosphorylation can influence decisions between recycling versus degradation and signaling was first described for β_2_AR and EGFR. EGFR activation by TGFα led to sustained MAPK activation and retrieval/recycling of the receptor, whereas activation by EGF induces fast receptor degradation and a transient MAPK activation (189). The SNX27-retromer retrieval subdomain allows to terminate G protein-coupled parathyroid hormone receptor signaling, a key regulation as shown by the deleterious effect of its constitutive activation on bone formation observed in *Snx27* deficient mice (190).

By controlling the residency time of internalized IFNAR complex in the endosome, the retromer is directly implicated in the fine tuning of JAK-STAT signaling duration and downstream transcription outputs (**Figure 3**). Indeed, a significant upregulation of genes known to be dependent on IFN stimulation was observed in VPS35 depleted cells upon IFN-α/-β activation, suggesting an aberrant prolonged activation of the JAK/STAT pathway (21). Thus, this study establishes a direct link between retromer-mediated sorting and modulation of intracellular signaling and gene transcription. In this context, a recent study proposed that the long-term effects of type I IFN could be explained by the persistence of receptor bound IFN-α2 inside endosomes. Endosomal IFN could even continue to signal from this compartment for days when the IFN-IFNAR complex negative regulators ISG15 or USP18 were missing (191). The endosome is therefore a crucial sorting station where a concerted choreography between Hrs/STAM and retromer complexes sequentially control the initiation and the termination of IFN-induced JAK/STAT signaling.

## Discussion

Since their discovery more than 60 years ago, numerous studies have tried to unravel the mechanisms underlying the signaling activity of IFNs and their cognate IFNAR receptor. Until recently, these studies have mainly focused on the initiation of JAK/STAT signaling at the plasma membrane in a linear manner. IFNAR membrane trafficking has been much less studied and all the less so when it comes to understand the role of the intracellular journey of IFNAR and its signaling. In agreement with the dogma that has long prevailed for transmembrane receptors, IFNAR trafficking was seen as a simple way to terminate signaling by passively removing receptors from the PM away from IFNs. This simplistic picture has recently changed with the demonstration that IFNAR trafficking is tightly associated with the control of JAK/STAT signaling. Nevertheless, the characterization of IFNAR trafficking has only recently begun and further studies are clearly needed to better understand the role of each trafficking step in the final IFN signaling response.

While most studies have addressed these mechanisms using IFN-α2 as a ligand, how IFN-β can transduce distinct activities remains a challenging and unresolved question. This is also the case for the other human type I IFNs including the twelve subtypes of IFN-α, IFN-ϵ, IFN-κ, and IFN-ω. Their distinct structures and IFNAR binding affinities may be translated into a selective modulation of IFNAR trafficking characteristics that it would be interesting to relate to their specific activities. Distinct IFN affinities could determine distinct IFNAR endocytosis rate and potentially control when signal is terminated at the endosomal level. Modulations of these parameters would eventually adjust the signal duration for each IFN subtype. Therefore, the regulation of trafficking events may add another level of complexity and control of the IFN stimulation outcomes.

The importance of better understanding IFNAR trafficking is not restricted to IFNAR as it will probably establish new paradigms in the control of signaling by trafficking for cytokine and transmembrane receptors beyond the prototypical EGFR and GPCRs. The extremely dynamic nature of the endosomal network and the rapid movement of vesicles through the cytosol, make it challenging to follow receptors during their journey in live cells. In particular, it is not known whether IFN receptor complexes made from different IFN subtypes would be found in common or separate endosomal compartments. However, the recent and continuous improvements in live cell imaging such as super resolution microscopy and AI-based segmentation approaches will enable us to make substantial progress in the near future. No, the journey does not end here.

## Author Contributions

NZ, CV, CL, and CB wrote the review. All authors contributed to the article and approved the submitted version.

## Funding

This work was supported by institutional grants from the Curie Institute, INSERM, CNRS, and by specific grants from Agence Nationale de la Recherche grants ANR-11-LABX-0038, ANR-10-IDEX-0001-02, ANR-NANOSTAT-15-CE11-0025-01, and ANR-NanoGammaR-15-CE11-0025-01. NZ was supported by a PhD fellowship from Ministère de l’Enseignement Supérieur et de la Recherche, and Ligue Nationale contre le Cancer, and by the European Commission under the Seventh Framework Programme [grant agreement n° HEALTH-F2-2013-602222 (Athero-Flux)] and CB by a post-doctoral fellowship from Ligue Nationale contre le Cancer.

## Conflict of Interest

The authors declare that the research was conducted in the absence of any commercial or financial relationships that could be construed as a potential conflict of interest.
